# Regulation of microRNA expression and function by nuclear receptor signaling

**DOI:** 10.1186/2045-3701-1-31

**Published:** 2011-09-21

**Authors:** Zhihong Yang, Li Wang

**Affiliations:** 1 Departments of Medicine and Oncological Sciences, Huntsman Cancer Institute, University of Utah School of Medicine, Salt Lake City, UT 84132, USA

**Keywords:** miRNAs, nuclear receptors, gene regulation

## Abstract

MicroRNAs (miRNAs) are small non-coding RNA transcripts that affect various cellular pathways by serving as regulators of gene expression at the translational and transcriptional level. Nuclear receptors (NRs) are ligand-activated transcription factors that regulate gene transcription by binding to the promoter region or by interacting with other transcription factors. NRs can regulate miRNA expression either at the transcriptional level, or through posttranscriptional maturation by interacting with miRNA processing factors. This review will summarize recent advances in knowledge of the modulation of miRNA expression by NRs. Increased understanding of the molecular basis of miRNA expression may enable new therapeutic interventions that modulate miRNA activities through NR-mediated signaling.

## Introduction

The binding of microRNA (miRNA, or miR) to the 3'-untranslated region of target mRNAs causes transcript degradation or interferes with translation initiation. This posttranscriptional inhibitory mechanism is of critical importance in fundamental cell processes, including development [1], proliferation [2], survival and death. During the past decade, much effort has been focused on elucidating the mechanism of miRNA target gene regulation, however, relatively little is known about the regulation of miRNA genes themselves [3]. A number of large-scale expression profiling studies have shown that the expression of miRNAs is dysregulated under various pathological conditions. Many miRNAs are expressed in a tissue-specific or developmental-stage-specific manner, thereby contributing greatly to cell-type-specific profiles of protein expression [4,5]. Growing evidence suggests that miRNAs can be regulated extensively at the levels of promoter transcription, methylation, miRNA processing, RNA editing, and miRNA-target interactions [6]. Transcriptional regulation by nuclear receptors is the primary level of control for miRNA expression (Table 1). Elucidation of the underlying mechanisms is crucial to understanding the pathways governing the miRNA network [7].

**Table 1:** miRNAs regulated by NRs

miRNA name	Regulation	note	target	**Ref**.
Let 7 family	ER;PPAR;PR	induced by estradiol; PPARα and PR agonist regulate let-7c.	K-ras; HMGA2; caspase-3; c-Myc; PGRMC1	[28,51,57,88-94]

miR-17-92 cluster	ER	c-Myc, adiol, binds induced by estr to the miR- 17-92 promoter; p53 and STAT3 bind to the miR-17-92 promoter.	Myc;E2F;HNF1;PTEN;B IM;ER;AIB1;cyclin D1	[30,88,95,96]

miR-21	ER;AR	STAT3, NF-κB, CREB and CBP/p300, ER and AR bind to the miR-21 promoter.	Pdcd4;PTEN;PPARα;	[28,97-101]

miR-221/222	ER;AR	NF-κB, c-JUN, ER and AR bind to the miR-221/222 promoter.	P27kip1;PTEN;ERα;PUM A	[29,45,102-106]

miR-200 family	ER;AR;PR	upregulated by estradiol, androgen and progesterone.	ZEB1; ZEB2; BMI1	[38,50,107]

miR-146a	ER;AR	repressed by estradiol, androgen and LPS; LPS induces NF-κB binding to the miR-416a promoter.	ROCK1; TRAF6; IRAK1; BRCA1; CD40L; STAT1	[39,108-112]

miR-26a	ER;AR	Estradiol induces miR-26a, which reduces PR mRNA level	EZH2;MTDH	[113,114]

miR-101	AR	ARE identified in the miR-101 promoter	EZH2; MAGI-2; MKP-1; ATP5B; COX-2; MYCN	[41,115-120]

miR-125b	AR	AR loading to the 5' UTR region	MUC1; PIGF; IGF-II; FGFR2; P53;PUMA;E2F3	[121-126]

miR-122	HNF4α	HNF4α binds to the miR-122 promoter.	Hfe; Hjv; CPEB; HCV; CAT-1; Smarcd1/Baf60α	[55,127-131]

miR-29a	FXR	FXR-responsive element in the miR-29a promoter; regulated by TGF-β, c-Myc, Hedgehog or NF-κ B	Ski; MCT1; PTEN; CDK6	[62,63,132-135]

miR-210	RARα/RXRα	RARα/RXRα heterodimers bind to the miR-210 promoter	FGFRL1; HOXA3; E2F3; RAD52	[70,136-138]

miR-23a/24-2	RARα/RXRα	RARα/RXRα heterodimers bind to the miR-23a/24-2 promoter	Runx2; XIAP; IL6R	[70,139-141]

miR-9	TLX	TLX binds to the downstream of miR-9 and miR-9 targets TLX mRNA to form a feedback loop	PDGFR-β; Nr2e1; FoxP1; Gsh2; NFκB1	[72,142-145]

miR-34a	FXR;SHP	p53 binds to the miR-34a promoter; FXR interacts with p53 through SHP to regulate miR-34a	FoxP1; Bcl-2;CDK4;E2F3; N-MYC;SIRT1;HMGA2	[59,146,147]

miR-433/127	SHP	SHP inhibits ERRγ which binds to the miR-433/127 promoters	HDAC6;BCL6	[80,148,149]

miR-206	SHP	SHP represses ERRγ leading to decreased YY1 which inhibits AP1 activation of the miR-206 promoter	Notch3; HDAC4; KLF4; Pax7	[81,82,150-153]

## 1.1 microRNA

miRNAs comprise a class of short (approximately 19-24 nucleotides) single-stranded non-coding RNAs that regulate gene expression through post-transcriptional mechanisms [8]. Since the discovery of RNA interference (RNAi) in 1993, efforts to identify endogenous small RNAs have led to the discovery of thousands of miRNAs in different species [9,10]. The newest database contains 16772 entries representing hairpin precursor miRNAs and expressing 19724 mature miRNA products in 153 species http://www.mirbase.org. Most of the miRNAs are conserved in closely related species and many have homologs in distant species, suggesting that their functions could also be conserved [11]. Accumulating evidence indicates that miRNAs play a central role in controlling a broad range of biological activities including embryonic development, cell proliferation, metabolic homeostasis, and apoptosis [12]. According to their locations in the genome, miRNA genes are classified into intragenic and intergenic regions. Although some intronic miRNAs are reported to have their own promoters, a significant percentage of miRNAs are embedded within introns or exons of protein coding genes and share the same transcriptional control of the host gene [13]. Mirtron is a notable exception that is spliced out of the host transcripts into the direct substrate of Dicer [14-16].

The biogenesis of miRNA starting in the nucleus is found to be quite complex, involving a vast group of protein complexes [17]. In general, miRNA genes are transcribed by Polymerase II as primary-miRNAs (pri-miRs). These large RNA precursors, which are several hundred base pairs in length, are often capped, spliced, and polyadenylated, and can encode sequences for multiple miRNA genes [6]. These precursors are processed by the RNAse-III-type endonuclease Drosha in association with the DiGeorge syndrome critical region gene 8 (DGCR8) (or Pasha in Drosophila, PASH-1 in Caenorhabditis elegans) into hairpin-shaped stem-loop structures of approximately 60-70 nt named pre-miRNAs. The pre-miRNAs are then exported out of the nucleus to the cytoplasm by Expertin-5, a Ran-GFP dependent transporter that specifically recognizes dsRNAs. In the cytoplasm, the pre-miRNAs undergo further processing by a second RNase III enzyme, named Dicer, into a miRNA-miRNA* duplex of variable length (~18-25 nt). Only one strand of the miRNA duplex, designated as the "guide" strand, is preferentially loaded into a large multi-protein miRNA ribonucleoprotein complex (miRNP, also referred to as the miRISC complex), and is used to modulate target gene expression. The "passenger" strand of the miRNA-miRNA* duplex (referred to as miRNA*) is excluded from the miRNP complex and degraded.

The well-known "seed" region, positions 2-8 nt from the 5'miRNA, is extremely important for miRNA targeting [18,19]. Depending on the degree of complementarity to the target mRNA, the mechanism of silencing target mRNA expression will be one of the following: 1) if there is perfect complementarity to a target mRNA, there will be target mRNA cleavage; and 2) in the case of incomplete complementarity, translational repression or alteration of mRNA stability will occur [20]. Bioinformatical prediction is based on the degree of seed region match with the 3'UTR of target genes. Furthermore, other "non-canonical" miRNA-mediated mechanisms of mRNA expression modulation are emerging [21-23]. Some miRNAs can bind to the open reading frame or 5'UTR of target genes; moreover, they have been shown to activate, rather than to inhibit, gene expression [21,24,25].

## 1.2 microRNA regulation by nuclear receptors

### 1.2.1 Nuclear receptors

Nuclear receptors (NRs) are ligand-activated transcription factors that regulate the expression of target genes by binding to cis-acting DNA sequences. The superfamily of nuclear receptors contains 48 human members that include classical receptors, adopted orphan receptors and orphan receptors. Classical receptors are regulated by an extensively studied group of endocrine ligands, such as the estrogen receptor (ER), androgen receptor (AR), progesterone receptor (PR), and glucocorticoid receptor (GR). Orphan nuclear receptors, like small heterodimer partner (SHP), have no natural ligands, and they behave like normal transcription factors. In the past few years, a class of so-called "adopted" orphan receptors (for which either natural or synthetic ligands have been identified) has arisen, such as peroxisome proliferator-activated receptors (PPARs) and liver × receptors (LXRs). A typical nuclear receptor usually contains five functional regions: the A/B region that contains an N-terminal activation function-1 (AF1) domain, the central C region that contains a DNA-binding domain (DBD), the C terminal E region that contains a ligand-binding domain (LBD) and activation function-2 (AF-2) domain, and the D hinge region that links the DBD to the LBD. Interestingly, the nuclear receptors Dax-1 and SHP only have LBDs, but they can interact with other transcription factors and function as corepressors in regulating their target genes. Nuclear receptors can activate or repress target genes by binding directly to DNA response elements as homo- or heterodimers, or by binding to other classes of DNA-bound transcription factors. Two groups of regulators, coactivators and corepressors, are recruited by NRs and in turn regulate the expression of downstream target genes.

### 1.2.2 Estrogen receptor (ER)

Since miRNAs are encoded by genes that are mainly transcribed by RNA polymerase II, their transcription can be regulated by a variety of transcription factors including NRs [26]. After estrogenic activation, ERs mediate transcription by interacting directly with specific estrogen response elements (EREs) located in the promoter/enhancer region of target genes, followed by recruitment of additional cofactors that have either activator or repressor functions on target genes [27]. For example, ERα binds directly to the promoter region of miR-221/222 and recruits NCoR and SMRT to suppress miR-221/222 expression [28]. The miR-221/222 may play a role in tamoxifen resistance because they have high expression levels in tamoxifen resistant breast cancer. In addition, c-Myc, induced by estrogen, can bind to the miR-17-92 locus in an estrogen-dependent manner [29]. E2 induces the expression of let-7 family members, as well as other miRNAs including miR-98 and miR-21, which reduce the levels of c-Myc and E2F2 proteins [30].

Apart from regulating the expression of miRNAs at the transcriptional level, ERα appears to be able to regulate the biogenesis of miRNAs. Drosha is directly inhibited by ERα [31]. Exportin 5, which controls the translocation of precursors, is induced by estradiol and progestins [32]. The expression levels of Dicer are induced by estradiol and progestins and are higher in ERα positive versus negative breast cancers [33,34]. Ago2, a component of RISC, is induced by estradiol. Ago1 and Ago2 are low in ERα positive breast cancers [33].

### 1.2.3 Androgen receptor (AR)

AR is a ligand-dependent transcription factor that regulates the expression of androgen target genes. Several miRNAs have been implicated in prostate cancer (CaP) development, including miR-125b [35], miR-21, miR-10a, miR-141, miR-150*, and miR-1225-5p [36,37], miR-205 and miR-200c [38], miR-146a [39], miR-221 and miR-222 [40], miR-101 and miR-26a [41], and the miR-15a-miR-16-1 locus [42]. Transfection of synthetic miR-125b, miR-21 or miR-141 stimulates androgen-independent growth of CaP cells [43], while miR-146a markedly reduces cell proliferation, invasion, and metastasis [39]. The expression of miR-125b, an androgen induced miRNA, is high in malignant prostate tissues. The miR-15a/miR-16-1 locus was homozygously deleted in a subset of prostate cancers leading to the abolishment of miR-15a, but not miR-16, expression [42]. The recruitment of AR to the 5'DNA region of miR-125b and miR-21 has been confirmed by ChIP analysis [37,43].

ARs can also bind to the promoter region of miR-221 to repress miR-221 expression in LNCaP cells [44]. Knocking-down miR-221 increases LNCaP cell migration and invasion by targeting DVL2 [45]. The circulating level of miR-21, miR-141 and miR-221 in the bloodstream might be useful as a prognostic marker in patients with prostate cancer [46,47]. miR-616 is over-expressed specifically in malignant prostate tissues, not in benign prostate specimens. Stable miR-616 overexpression in LNCaP cells stimulates prostate cancer cell proliferation and castration resistance [48].

### 1.2.4 Progesterone receptor (PR)

In mammalian pregnancy, uterine quiescence is maintained by elevated circulating progesterone (P4) acting via PR. The miR-200 family, including miR-200b/c/429 and miR-200a/141, is upregulated during late gestation and labor [49]. P4 injection causes a modest decrease in myometrial expression of miR-200b/429, yet it also significantly increases ZEB1 mRNA and protein, a target of the miR-200 family. Further studies show that ZEB2 rather than ZEB1 is acting as a transcriptional repressor on the miR-200c/141 promoter [50]. In SKOV-3 cells, the expression of let-7, which targets PGRMC1 (progesterone receptor membrane component 1), is increased after stimulation with progesterone [51]. Progesterone is also reported to regulate miR-320 expression [52]. Conversely, miR-126-3p inhibits PR protein expression as well as the proliferation of mammary epithelial cells by targeting the PR 3'UTR directly [53].

### 1.2.5 Hepatocyte nuclear factor-4α (HNF4α)

HNF-4α is a highly conserved nuclear receptor that is expressed in the liver, kidney, intestine, and pancreas. HNF-4α is a key regulator of energy metabolism, glucose and lipid homeostasis [54]. A putative binding site for HNF-4α is found in the conserved core element of the hpri-miR-122 promoter. The miR-122 promoter activation by HNF-4α is further enhanced by the addition of PGC1α, a well-recognized co-activator of HNF4α [55].

### 1.2.6 Peroxisome proliferator-activated receptor (PPAR)

MiR-29a and 29c levels are decreased in the rat heart after 7-day treatment with PIO, a peroxisome proliferator-activated receptor (PPAR)-γ agonist [56]. Wy-14,643, a specific PPARα agonist, inhibits microRNA let-7c expression via a PPARα-dependent pathway. The lack of any significant difference in basal let-7c expression between the WT and PPARα-null mice suggests an active transrepression mechanism, where the receptor is recruited to the genomic regulatory region of let-7c following ligand treatment [57]. Because let-7c targets the c-Myc 3'UTR for degradation, the PPARα-mediated induction of c-Myc via let-7c subsequently increases the expression of oncogenic miR-17-92 clusters [58].

### 1.2.7 Farnesoid × receptor (FXR)

FXR is the primary biosensor for endogenous bile acids and regulates the expression of numerous genes involved in lipid and glucose metabolism. miRNAs regulated by FXR were detected by miRNA microarray analysis with hepatic RNAs of wild type or FXR-null mice. Of the miRNAs tested, the level of miR-34a is upregulated in FXR-null mice. The mechanism of this regulation is as follows: activation of FXR induces SHP, which in turn suppresses miR-34a gene transcription by inhibiting p53 binding to the miR-34a promoter [59]. Treatment with GW4064, a synthetic FXR ligand, upregulates miR-29a in hematopoietic stem cells (HSCs) isolated from wild-type mice, rats, and humans but not from FXR-null mice. A FXR-responsive element has been identified in the miR-29a promoter, which is involved in the regulation of extracellular matrix (ECM) production in liver [60]. The expression of miR-29a is also negatively regulated by TGF-β, c-Myc, hedgehog or NF-κB signaling in liver and lung fibrosis [61-63].

### 1.2.8 Liver × receptor (LXR)

LXR plays an important role in the metabolism and homeostasis of cholesterol, lipids, bile acids, and steroid hormones [64]. After endogenous or synthetic ligand binding, LXR forms a heterodimer with retinoid × receptor (RXR) and binds to LXR response elements (LXREs) in the promoters of LXR target genes. Treatment with GW3965, a LXR ligand, induces the expression of mature hsa-miR-613 in primary human hepatocytes as well as in human hepatoma HepG2 and Huh7 cells. The positive regulation of hsa-miR-613 by LXR is mediated by the sterol regulatory element binding protein (SREBP)-1c, a known LXR target gene [65]. Interestingly, hsa-miR-613 can target the 3'UTR of endogenous LXRα. The negative regulation mediated by hsa-miR-613 and SREBP-1c and the previously reported positive regulation mediated by an LXRE constitute a negative autoregulatory feedback loop to ensure a tight regulation of LXRα [66].

### 1.2.9 Pregnane × receptor (PXR)

PXR is a crucial regulator of drug metabolism in liver and small intestine. PXR dimerizes with RXRα and binds to the response elements of its target genes, including CYP3A4 [67]. Eleven out of the three hundred human miRNAs that have been identified exhibit a more than three-fold altered expression in HepG2 cells treated with the PXR ligand rifampicin. MiR-31 is 5.4-fold down-regulated, whereas all others, namely miR-193a -5p, miR-296-5p, miR-324-5p, miR-379, miR-411, miR-489, miR505, miR-519a, miR-545 and miR-548b-5p, exhibit up to 5.8-fold higher expression levels [68,69]. Interestingly, miRNA can regulate PXR expression too. For example, miR-148a post-transcriptionally regulates human PXR, resulting in the modulation of the inducible and/or constitutive levels of CYP3A4 in human liver [67].

### 1.2.10 Retinoic acid receptor (RAR) and retinoic × receptor (RXR)

To identify potential transcription factor binding sites (TFBS) in the promoter regions of 247 human intergenic microRNAs (from a miRNA promoter dataset provided by Mahony et al [70]), NHR-Scan was used for the PML-RARA fusion protein [69]. Sixty-five microRNA promoters contain a PML-RARA predicted site. Further investigations are focused on the miR-210 and cluster of miR23a/24-2. After treating with the RAR agonist all-trans retinoic acid (ATRA), expression of miR-23a and miR-210 is increased. ChIP experiments directed against both RARα and RXRα in 293T cells show that RARα/RXRα heterodimers bind miR-210 and miR-23a/24-2 promoters. This method which is start with prediction of NR binding site in miRNA promoter database, is different from other microRNA array first analysis [69].

### 1.2.11 Nuclear receptor TLX (homologue of the Drosophila tailless gene)

Orphan nuclear receptor TLX, which is expressed in the neuroepithelium of the embryonic mouse brain and in adult neurogenic regions, is essential for neural stem cell (NSC) proliferation [71]. TLX binds to downstream of the miR-9 sequence at the miR-9-1 locus and represses miR-9 at the transcriptional level. Meanwhile, miR-9 targets TLX mRNA for destabilization and/or translational inhibition, reducing TLX protein levels. MiR-9 and TLX thus form a feedback loop to regulate the switch of neural stem cell proliferation and differentiation [72].

### 1.2.12 Small heterodimer partner (SHP)

SHP is an orphan nuclear receptor that contains the ligand-binding domain (LBD), but lacks the DNA binding domain (DBD) [73]. SHP functions as a transcriptional repressor though interacting with other NRs, and plays important roles in several metabolic diseases and in liver carcinogenesis [74-77]. SHP interacts with ERRγ to control the expression of miR-433 and miR-127 in several mammalian species [78-80]. SHP also activates miR-206 expression through a ''dual inhibitory'' mechanism [81], which in turn targets Notch3 for degradation [82]. The SHP/FXR signaling is important in controlling the expression of miR-34α and its target SIRT1 [83].

### 1.2.13 Other NRs

Other members of the NR family are also involved in miRNA expression regulation. Glucocorticoids (GC) bind to both the glucocorticoid receptor (GR) and mineralocorticoid (MR). Both receptor types act through transactivation at glucocorticoid response elements (GREs) [84]. GR controls a variety of physiological functions, such as metabolism, development, and reproduction; whereas MR is critical for controlling sodium and potassium transport, pathophysiology of hypertension, and cardiac fibrosis [85]. Several miRNAs/mirtrons are regulated by GC in patients and cell lines, including the myeloid-specific miR-223 and the apoptosis and cell cycle arrest-inducing miR-15/16 clusters. miR-15b/16 increases GC sensitivity in leukemia cell lines, further suggesting that miRNA regulation is a vital component of GC signaling [86]. In addition, miR-208 potentiates βMHC expression through a mechanism involving the thyroid hormone receptor [87].

## 1.3 Conclusion

**Figure 1:**
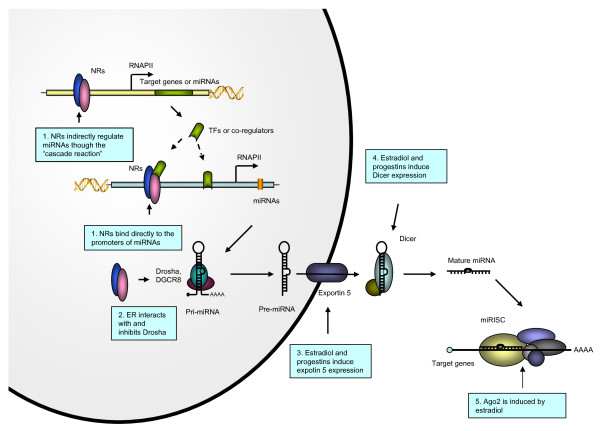
**Nuclear receptor regulation of miRNA expression and biogenesis**. 1. The miRNA genes are transcribed by RNA polymerase II to produce pri-miRNAs. NRs bind directly to the miRNA gene promoters or interact with other transcription factors that bind to the target miRNA promoters. 2. The pri-miRNAs are cleaved by Drosha and DGCR8 to become pre-miRNAs, which have hairpin-shaped stem-loop structures. ERα inhibits Drosha by direct interaction. 3. The pre-miRNAs are exported to the cytoplasm by Expertin-5 that is induced by estradiol and progestins. 4. The pre-miRNAs undergo further processing by Dicer to become mature miRNAs. The expression of Dicer is increased by estradiol and progestins. 5. The mature miRNAs are loaded to the miRISC to regulate target gene expression. Ago2, a component of the RISC, is induced by estradiol.

NRs appear to regulate miRNA expression via three means: direct binding to the promoter regions of miRNAs, indirect regulation of miRNA expression through NR target genes, and involvement in regulation of miRNA biogenesis (Figure 1). Microarray analyses has revealed large numbers of miRNAs that are differentially regulated by NRs or their ligands, but the detailed regulatory mechanisms remain to be elucidated. Future exploration of the interactions between NRs and miRNAs in the regulation of gene expression networks is needed for better understanding of miRNA modulation and function by NR signaling.

## Competing interests

The authors declare that they have no competing interests. All authors read and approved the final manuscript.

## Authors' contributions

ZY prepared the draft, LW made the final version. All authors read and approved the final manuscript.
